# Succession of the yellow tang (*Zebrasoma flavescens*) microbiome from hatch to settlement in a recirculating aquaculture system

**DOI:** 10.7717/peerj.21690

**Published:** 2026-09-21

**Authors:** Annie R. Deck, Sarah J. Tucker, Brianna M. Hauser, Matthew J. Iacchei, Chatham K. Callan, Michael S. Rappé, Olivia D. Nigro

**Affiliations:** 1 School of Ocean and Earth Science and Technology, Hawaiʻi Institute of Marine Biology, University of Hawaiʻi at Mānoa, Kāneʻohe, HI, United States of America; 2 Marine Biology Graduate Program, University of Hawaiʻi at Mānoa, Honolulu, HI, United States of America; 3 Department of Marine Science, College of Natural and Computational Sciences, Hawaiʻi Pacific University, Waimānalo, HI, United States of America; 4 Helmholtz Institute for Functional Marine Biodiversity, Oldenburg, Germany; 5 Helmholtz Centre for Polar and Marine Research, Alfred Wegener Institute, Bremerhaven, Germany; 6 Marine Biological Laboratory, Josephine Bay Paul Center for Comparative Molecular Biology and Evolution, Woods Hole, MA, United States of America; 7 College of Agriculture, Forestry & Natural Resource Management, Pacific Aquaculture and Coastal Resources Center, University of Hawaiʻi at Hilo, Hilo, HI, United States of America

**Keywords:** Aquaculture, 16S rRNA sequencing, *Vibrio*, Yellow tang, Microbiome, Larval microbiome development

## Abstract

Ornamental fish aquaculture is receiving increasing scientific attention as a sustainable means of meeting demand for reef species from the aquarium trade. However, low juvenile recruitment in an aquaculture environment remains a key industry bottleneck for many high-demand pelagic-spawning species. Here, the microbiome of a yellow tang (*Zebrasoma flavescens*) larval cohort from hatch to the juvenile stage (76 days post-hatch) in a recirculating aquaculture system was investigated using a high throughput 16S rRNA gene amplicon sequencing approach. The taxonomic composition of microorganisms was assessed from the gastrointestinal tract of larval yellow tang, their feed, and the recirculating rearing water at eight key developmental timepoints. We observed a dynamic larval microbiome that displayed developmental stage-specific signatures. While some microbial taxa were ubiquitous throughout the larval stage (*i.e.*, Proteobacteria), others, including Fusobacteriota, were restricted to specific growth stages. In addition to the role of ontogeny on host microbiomes, shifts in the microbial communities associated with feed types were found to coincide with pronounced changes in specific bacterial genera obtained from larval-derived microbiomes. Notably, in larval samples the relative abundance of the genus *Vibrio* peaked at 36 and 55 days post-hatch (97.2% and 94.2%, respectively), coinciding with the addition of enriched *Artemia franciscana* brine shrimp into their diet. The introduction of *Vibrio* species from prey cultures, specifically with *Artemia*, highlights that feed-associated microbiomes may play a substantial role in determining host microbiomes in early life, and illustrates the importance of further understanding the nature of *Vibrio* species present in food items used in an aquaculture setting.

## Introduction

Aquaculture offers a sustainable option to reduce aquarium trade-driven fishing pressure on wild populations of marine ornamental fish (Palmtag, 2017; Calado, 2017). However, the captive-rearing of marine ornamentals has not kept pace with demand. Currently, only ∼4.2% of ornamental species are produced commercially (Sweet & Pedersen, 2019; Holcombe et al., 2022), with the majority being freshwater species. As a result, 90% of the marine ornamental fish in the aquarium trade are sourced directly from wild populations (Palmtag, 2017). This ongoing reliance on wild collection has raised concerns regarding both the sustainability of collection practices globally (Chen et al., 2020; Olivotto et al., 2017), as well as the potential declines in wild fish stocks (Williams et al., 2009) that provide valuable ecosystem services such as algal grazing (Holbrook et al., 2016).

In Hawaiʻi, the yellow tang (*Zebrasoma flavescens*) has historically been one of the most heavily traded ornamental species (Williams et al., 2009; DLNR, 2015). In recent years, yellow tang collection in Hawaiian waters has comprised over 80% of the ornamental fishery in the state by volume and revenue (DLNR, 2015). This high demand and economic importance support the continued development of commercial aquaculture production for the species. Yellow tang were first successfully cultured in an aquaculture setting in 2015, a major achievement for the ornamental aquaculture field (Holt et al., 2017; Callan et al., 2018). However, commercial-scale production remains constrained by persistently high larval mortality rates of approximately 99% from hatch to the juvenile stage (Holt et al., 2017). Low juvenile recruitment represents a major bottleneck not only for yellow tang, but also for many other pelagic-spawning ornamental species (Chen et al., 2020), making reductions in the rates of early mortality critical for advancing commercial ornamental fish production (Olivotto et al., 2017).

The challenges of culturing yellow tang are partly attributable to their life history. As is characteristic of pelagic-spawning teleosts, larvae hatch with underdeveloped physiologies, including a rudimentary gastrointestinal tract and a sealed mouth (Holt et al., 2017). Newly hatched larvae rely entirely on endogenous nutrition from their yolk sac for the first 2 to 3 days post-hatch (dph), after which they transition to exogenous feeding (Olivotto et al., 2005). This transition is marked by rapid morphological changes, including opening of the mouth, increases in mouth gap, and maturation of the gut (Burgess & Callan, 2018). While the diet of *Acanthuridae* larvae in the wild is poorly understood, first-feeding larvae are believed to consume small prey such as copepod nauplii and pteropods (Llopiz & Cowen, 2009; Burgess & Callan, 2018). In an aquaculture setting, overlapping periods of varying size classes of live prey including copepods, rotifers, and brine shrimp have been successful in rearing pelagic-spawning species like the yellow tang (Holt et al., 2017; Callan et al., 2018) and the Pacific blue tang (*Paracanthurus hepatus*) (DiMaggio et al., 2017). Moreover, as larvae continue to grow from the pre-flexion to flexion stage (8–13 dph) and mouth gap widens, progressively larger prey items such as rotifers are introduced. Development of a caudal fin (∼15 dph) marks a significant milestone enabling the transition from passive to active swimming and hunting behavior and coincides with the addition of newly hatched *Artemia* to their diet. Later larval stages, including the acronurus phase (∼55 dph), allow for larger *Artemia* to be included as feed before the larvae settle as juveniles. In the wild, settlement is characterized by a shift from a pelagic to benthic reef-dwelling lifestyle (Holt et al., 2017; Callan et al., 2018).

The critical developmental transition to exogenous feeding, differentiated by a peak in larval mortality (estimated 25–50% loss of the cohort) (Holt et al., 2017; Callan et al., 2018), occurs concurrently with gut maturation and the initial seeding and diversification of the gut microbiota (Egerton et al., 2018). The health of vertebrate animals is critically influenced by microbial communities colonizing both internal and external host surfaces, with the gut microbiome being particularly important (Turnbaugh et al., 2007; McFall-Ngai et al., 2013). Consisting of bacteria, archaea, viruses, and fungi, the gut microbiome has been extensively studied in humans (Turnbaugh et al., 2007) and model species such as the zebrafish (*Danio rerio*) and mice (*Mus musculus*) (Goodman et al., 2011; Roeselers et al., 2011; Rawls, Samuel & Gordon, 2004; Luan et al., 2023). These studies have demonstrated the gut microbiome’s vast metabolic potential and essential roles in digestion, nutrient acquisition, and immunity against opportunistic pathogens (Turnbaugh et al., 2007; Llewellyn et al., 2014; Talwar et al., 2018).

The gut microbiome has also been increasingly recognized as an important factor in aquaculture production (Egerton et al., 2018; Perry et al., 2020). In zebrafish, the gut microbiome contributes to vital lipid metabolism and the development of innate immune system during early life stages (Rawls, Samuel & Gordon, 2004; Roeselers et al., 2011), and in commercially produced species like tilapia (*Oreochromis niloticus*) and Atlantic salmon (*Salmo salar*), has been linked to survival and growth performance (Giatsis et al., 2015; Llewellyn et al., 2014; Wang et al., 2022). Both dietary composition and developmental stage have previously been identified as influential drivers of gut microbiome development in larval fish (Stephens et al., 2016; Wilkes Walburn et al., 2018), suggesting that the dietary and morphological transitions characteristic of yellow tang larval development may play an important role in shaping their gut microbial communities. However, understanding of gut microbiomes in non-model teleost fish, particularly during larval developmental stages, remains limited (Llewellyn et al., 2014; Perry et al., 2020). Marine pelagic-spawning fish are especially understudied, creating a critical knowledge gap for aquaculture applications where insights into larval microbiome establishment could improve rearing protocols.

To explore the role of the microbiome in the development and survivorship of yellow tang larvae, we monitored a single cohort of yellow tang for 76 days from hatch to the juvenile stage of development. Microbiomes derived from larval yellow tang, food, and rearing water at each sampling timepoint were investigated using 16S rRNA gene amplicon sequencing and statistical analysis. This dataset provides unique insights into the microbiome development of a difficult-to-rear ornamental species in an aquaculture setting and can be used to inform larval rearing practices for yellow tang and other marine pelagic-spawning species.

## Materials & Methods

### Yellow tang husbandry and sample collection

On May 30, 2023, a single 1,000 L rearing tank at the Oceanic Institute’s hatchery was stocked with ∼40,000 fertilized eggs from a single spawning event of adult broodstock yellow tang (Callan et al., 2018). Sampling occurred from June 1 to August 13, 2023. Rearing conditions have been described in detail previously (Holt et al., 2017; Callan et al., 2018). Briefly, larvae were raised in a recirculating aquaculture system (RAS) featuring mechanical and biological filtration that incorporates 50 µm and 100 µm bag filters, a protein skimmer, ultraviolet light, a biofilter, and a bead filter. Representative environmental conditions for the rearing water ranged from 25–26 °C, a pH of 7.8–7.9, and salinity of 32 psu (Callan et al., 2018). Water quality parameters were held constant and monitored daily for the duration of this study. Due to their limited variability, these parameters were not evaluated as potential explanatory factors of differences in microbiome composition. Larval mortality was monitored periodically by visual inspection of the tanks. At the final sampling timepoint, surviving larvae were counted to calculate hatch-to-settlement survivorship.

### Feed types

**Figure 1:**
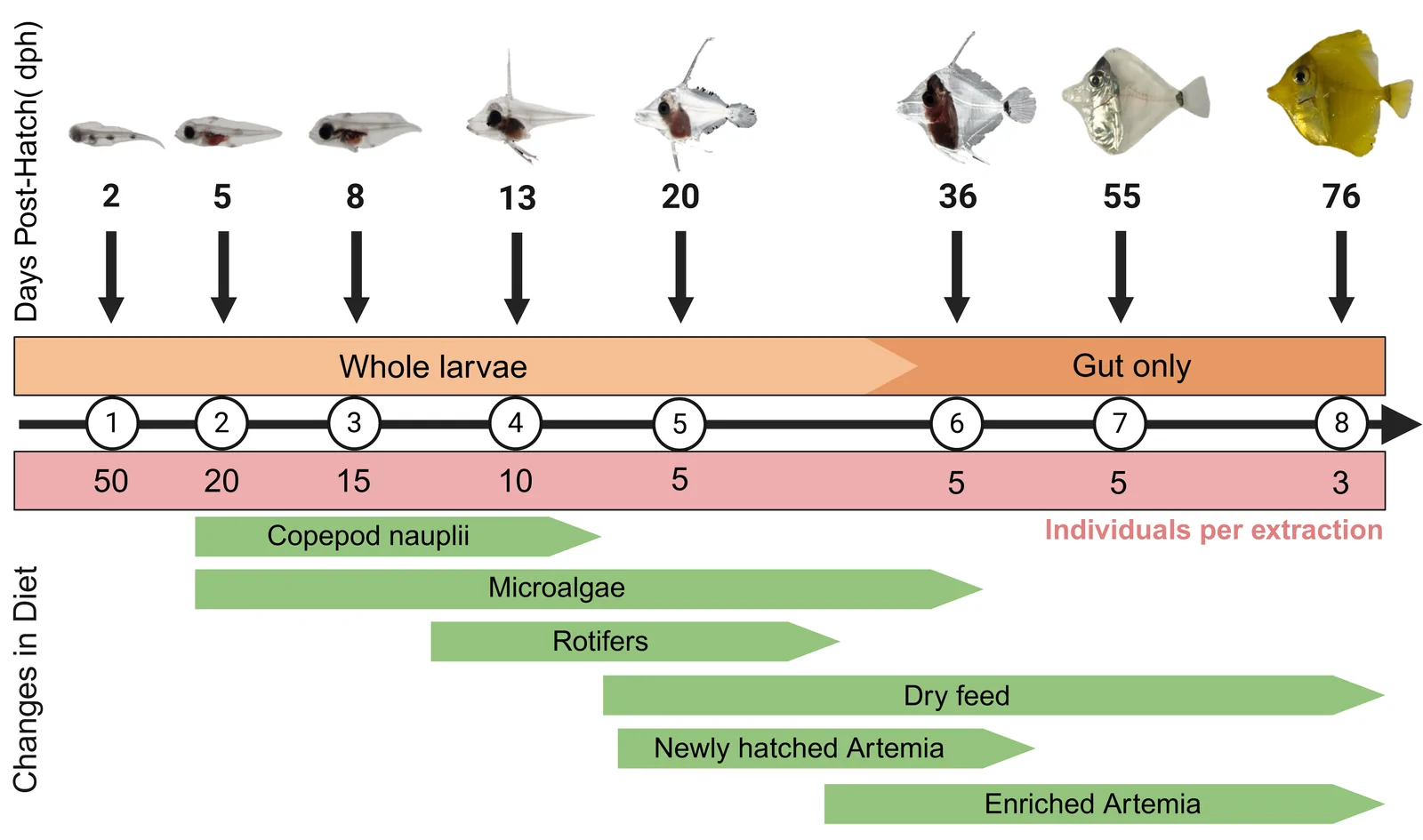
Schematic of experimental design. At each sampling timepoint (1–8) larvae, food, and tank water samples were collected. For larval samples, at timepoints 1–5 whole larvae were homogenized for extraction (light orange bar), while dissected guts were homogenized at timepoints 6–8 (dark orange bar). Number of larvae per extraction indicated within pink bar. Created in BioRender. Deck, A. (2026).

Throughout larval development, larvae were fed a mixed diet of live prey of increasing size classes supplemented with formulated dry feed (Fig. 1). All live prey and microalgae used in this study came from self-maintained cultures at the Oceanic Institute (Waimanalo, HI). Copepod nauplii (*Parvocalanus crassirostris*) were the first live prey offered as larvae transitioned from endogenous to exogenous feeding (approximately 3 dph). Concurrent with the introduction of copepods, microalgae (*Isochrysis galbana*) were added to the rearing tanks at a concentration of ∼3 × 10^5^ cells mL^−1^ and maintained through 36 dph to serve as a food source for the smaller size-classes of live prey (Callan et al., 2018). Larger live prey was introduced sequentially including rotifers (*Brachionus rotundiformis*) at 10 dph, newly hatched brine shrimp (*Artemia franciscana)* at 15 dph*,* and enriched brine shrimp (*A. franciscana)* at 30 dph. *Artemia* cultures were enriched with Larviva Multigain (BioMar, Aarhus, Denmark), an additive containing omega-3 and omega-6 fatty acids, vitamins, and the commercial probiotic Bactocell (Lallemand, Montreal, Canada). Bactocell contains a strain of *Pediococcus acidilactici*, a lactic acid bacterium reported to improve fish growth and immunity (Al-Hisnawi et al., 2019; Gauthier et al., 2019).

### Sampling

**Figure 2:**
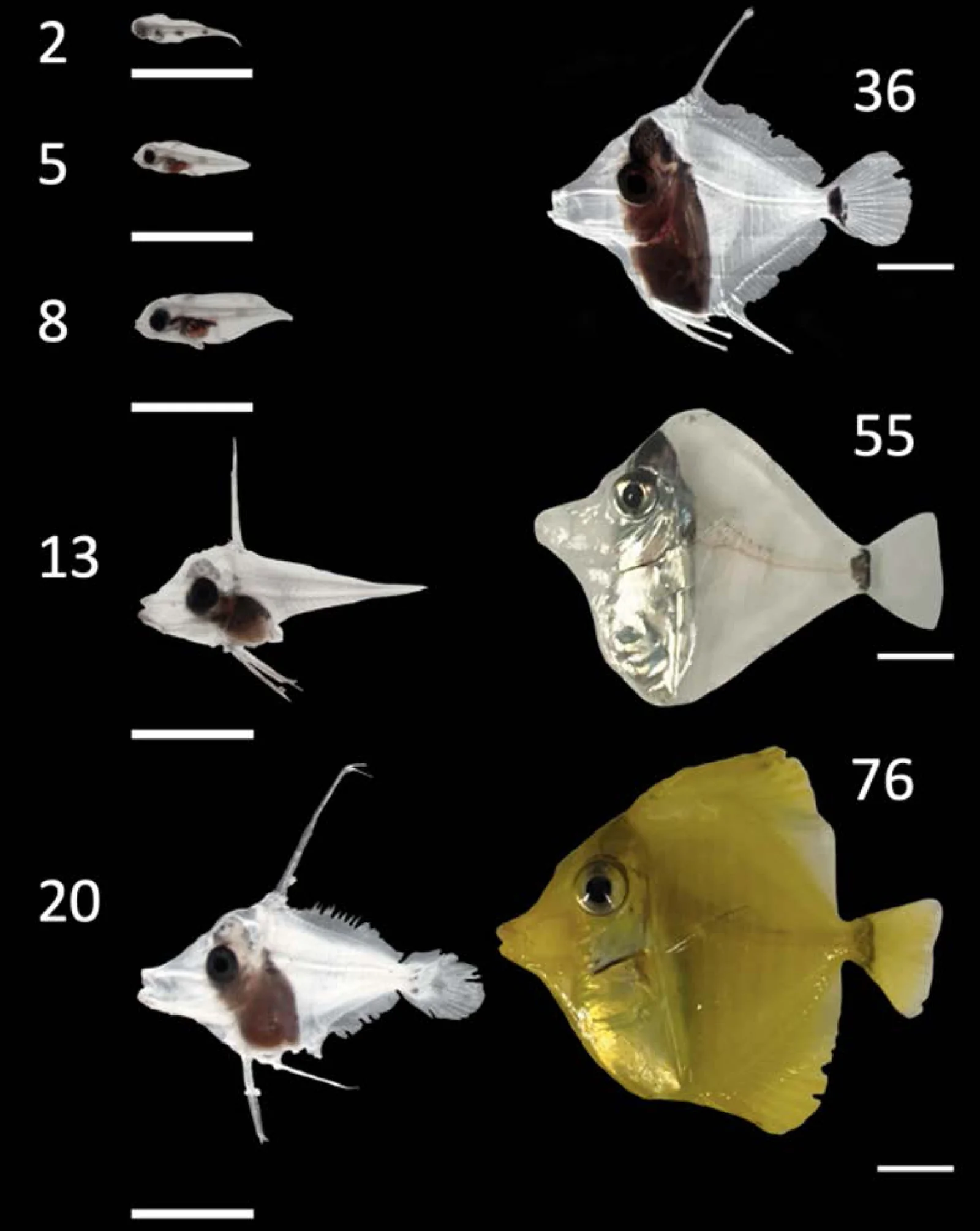
Larval yellow tang (*Zebrasoma flavescens.*) development from 2 days post-hatch (dph) to settlement (76 dph). Larval photos collected at each sampling event. Numbers indicate dph and scale bar indicates a length of one mm.

Eight sampling timepoints were strategically selected to capture major developmental milestones, such as transition from endogenous to exogenous feeding, as well as dietary transitions of the yellow tang from hatch to settlement (Figs. 1, 2). Specifically, the sampling timepoints captured (1) endogenous feeding larvae (2 dph); (2) larvae beginning to transition to exogenous feeding on copepod nauplii (5 dph); (3) larvae fully transitioned to exogenous feeding (8 dph); (4) larvae with rotifers introduced as feed to supplement the copepod nauplii (13 dph); (5) larvae with developed caudal fin and dry feed and newly hatched *Artemia* introduced into the diet (20 dph); (6) larvae with larger enriched *Artemia* introduced into the diet (36 dph); (7) larvae that have reached acronurus phase of development (55 dph); and (8) settled juvenile yellow tang (76 dph).

Four sample types were collected at each of the eight timepoints, including (1) whole larvae or dissected guts; (2) rearing water (particles >0.2 µm and <40 µm from rearing tank water); (3) tank particulate matter (particles >40 µm from rearing tank water); and (4) food stock (direct food samples prior to tank addition). Food stock samples were not collected at the first two timepoints (2 and 5 dph). Samples were collected in the morning approximately two hours after the first feeding of the day, when applicable. Larval samples were collected in triplicate at each time point to capture biological variability among host-associated microbiomes, while rearing water, tank particulate matter, and food stock samples were not replicated as these sample types served as a representative of the shared environmental or food stock pools within the single recirculating aquaculture system as opposed to independent experimental units. While larvae readily avoided collection during rearing water sampling, tank water and sample filters were nonetheless visually inspected to ensure larvae were not captured in this process. All materials used for sample collection and filtration were sterilized by acid rinse and autoclaving prior to use.

Tank particulate matter samples were collected by filtering ∼1 L of rearing tank water through a 40 µm sterile nylon mesh. By visual inspection, significant amounts of food were captured on the filter however, other detritus and fecal matter were also likely retained in this sample type. The nylon mesh was then vortexed in 800 µl of lysis buffer (QIAamp PowerFecal Pro DNA Kit lysis buffer CD1; Qiagen Inc., Germantown, MD) for 3 min to resuspend particles, and the resulting supernatant transferred to bead beating tubes for extraction. Filtrate from the tank particulate matter samples was subsequently filtered through a 0.2 µm pore-sized Sterivex filter (MilliporeSigma, Burlington, MA, USA), generating the corresponding rearing water sample. Sterivex filter membranes were removed from their casings as previously described (Cruaud et al., 2017) and placed directly in bead beating tubes containing 800 µl lysis buffer. Food stock samples consisted of 250 mg of food collected directly from the source stock. In cases where multiple food items were offered at a single time point, equal parts of each food type were used. The 250 mg samples were then suspended in 800 µL of lysis buffer in a bead beating tube.

Pools of yellow tang larvae were collected in triplicate at all timepoints using a sterile fine nylon net and counted using a SZ61 Stereo Microscope (Olympus, Center Valley, PA, USA). Specimens were collected from the tank at random with no pre-established criteria. A target of 250 mg of larval tissue per replicate pool was used for all extractions consistent with extraction kit manufacturer recommendations. For sampling during the first 5 timepoints (2–20 dph), approximately 250 mg of whole larvae were collected for downstream microbiome analyses as their small body size prohibited dissection of gut tissue (Wilkes Walburn et al., 2018). As larval body mass increased, the number of pooled larvae decreased across the remaining timepoints accordingly, from 50 individuals per replicate at timepoint 1 to 5 individuals per replicate at timepoint 5 (Figs. 1, 2). Following enumeration, larvae were euthanized by rapid chilling in accordance with procedures approved by Hawaiʻi Pacific University’s Institutional Animal Care and Use Committee (IACUC) (IACUC Protocol #A2223001). Mortality was visually confirmed by cessation of opercular movement for >20 min. Larval specimens were rinsed with 70% ethanol followed by three rinses with sterile water to reduce the presence of microbial communities from the skin, gills, and body surface. The entire larval pools were then homogenized, transferred to bead beating tubes, and suspended in 800 µL lysis buffer.

For timepoints 6 through 8 (36–76 dph), larval guts were aseptically dissected from five individuals per replicate pool for timepoints 6 and 7 and three individuals per replicate pool for timepoint 8 following previously published procedures (Wilkes Walburn et al., 2018). The gut tissue was then pooled and homogenized using a handheld Polytron PT 1200 E homogenizer (Kinematica, Bohemia, NY, USA) for approximately 5 min at high speed. A 250 mg subsample of homogenized gut was transferred to bead beating tubes and suspended in 800 µL lysis buffer. In total, 339 yellow tang specimens were collected, and none were excluded from analysis. Though the small size and underdeveloped physiology of yellow tang larvae during early life make isolation of gut tissue challenging compared to demersal spawners, the yellow tang aquaculture program at the Oceanic Institute offers a unique and valuable opportunity to get insight into the microbial dynamics of pelagic spawning species during early ontogeny.

### DNA extraction and sequencing

Following suspension in lysis buffer, samples were mechanically disrupted using the PowerLyzer 24 Homogenizer (Qiagen) at 2,000 rpm for 30 s. Microbial DNA was extracted with the QIAamp PowerFecal Pro DNA Kit (Qiagen) following manufacturer instructions. The DNA extraction methods used in this study were selected based on extensive testing for this system (Deck, 2024). DNA yield was quantified using the Qubit dsDNA BR (Broad-Range) and HS (High-Sensitivity) Assay Kits (Life Technologies, Foster City, CA, USA).

Microbiome DNA was amplified via polymerase chain reaction (PCR) targeting the V4 region of the 16S rRNA gene (Caporaso et al., 2011) using the Environmental Microbiome Project (EMP) primers 515F GTGYCAGCMGCCGCGGTAA (Parada, Needham & Fuhrman, 2016) and 806R GGACTACNVGGGTWTCTAAT (Apprill et al., 2015). The 20 µL reactions contained 0.2 µM of each primer, 10 µL 2x Platinum II Hot-Start PCR Master Mix (Invitrogen, Waltham, MA, USA), and 2 µL of template DNA. The PCR reaction included an initial step at 94 °C for 2 min followed by 30 cycles of 94 °C for 15 s, 54 °C for 15 s, 68 °C for 7 s, and a final extension at 68 °C for 3 min. A second PCR reaction was conducted to attach Nextera library indexes. The second 20 µL reaction included 0.5 µM of Nextera XT Index i7 and i5 (Illumina, San Diego, CA, USA), two µL of round 1 PCR product, and 10 µL 2x Platinum II Hot-Start PCR Master Mix. Cycling conditions for the second round of PCR included an initial denaturation step at 94 °C for 2 min followed by eight cycles of 94 °C for 15 s, 53 °C for 15 s, 68 °C for 15 s, and a final extension at 68 °C for 3 min. Following amplification and library preparation, samples were sequenced on an Illumina MiSeq v3 300 bp paired-end run at the Advanced Studies in Genomics, Proteomics, and Bioinformatics (ASGPB) facility at University of Hawaiʻi at Mānoa (Honolulu, HI).

### Bioinformatics and statistical analysis

Demultiplexed paired-end reads were truncated (forward, 260 bp; reverse, 220 bp), merged, denoised, and resolved into exact amplicon sequence variants (ASVs) using the DADA2 (v.2021.24) pipeline (Callahan et al., 2016) and taxonomically identified using the Silva v.138.1 database (Quast et al., 2013) in Qiime2 (v.2021.24) (Bolyen et al., 2019). To minimize the impact of sequencing artifacts, ASVs fewer than 10 reads across all samples or appearing in fewer than two samples were removed (Cao et al., 2021). ASVs that could not be taxonomically assigned at the domain or phylum level or that were identified as eukaryotic plastids were also removed.

Statistical analyses and data visualizations were conducted in RStudio v2022.12.0 using phyloseq (McMurdie & Holmes, 2013), ggplot2 (Wickham, 2016), and vegan (Oksanen et al., 2022) packages. For multivariate analysis across the entire dataset, dispersion within groupings was assessed using the ‘betadisper’ function and permutational multivariate analysis of variance (PERMANOVA) was implemented with the ‘adonis’ function. Hierarchical clustering was implemented with the SigClust2 function (Kimes et al., 2017) and used to further analyze community differences between developmental stages sampled.

For phylogenetic analysis, all exact ASVs assigned to the genus *Vibrio* were imported into the ARB software package (Ludwig et al., 2004). The sequences were aligned using SINA v.1.2.12 (Pruesse, Peplies & Glöckner, 2012) to create a curated database of all taxonomically described Vibrio species based on ‘The All-Species Living Tree’ Project version LTP_08_2023 (Ludwig et al., 2021). A phylogenetic tree was estimated by using the RAxML maximum likelihood method with the General Time Reversal (GTR) model of nucleotide substitution under the gamma and invariable models of rate heterogeneity (Stamatakis, 2006), using a sequence mask of 248 nucleotide positions.

## Results

### Larval development and survivorship

Throughout the experiment, yellow tang larvae underwent substantial physiological and developmental transformations (Figs. 1, 2). At the first sampling timepoint (2 dph), the larvae were unable to forage due to closed mouths and undeveloped eyesight and instead relied completely on their oil droplet for nutrition. By 5 dph, the majority of surviving larvae transitioned to exogenous feeding, marked by red gut coloration following consumption of their first prey item, copepod nauplii. By 8 dph, exogenous food was observed in 100% of surviving larvae, and the first signs of ventral fin development were observed. Mortality was visually estimated to be 50–75% at this timepoint, marking the end of the first week post-hatch. From 13 to 55 dph, larvae continued to exhibit osteological development including key milestones such as extension. During this period, the larvae transitioned from smaller prey like copepod nauplii to newly hatched *Artemia* and dry feed. By 55 dph, larvae reached their final feed transition to enriched *Artemia* and dry feed. At 76 dph, most individuals had attained characteristic yellow pigmentation and were classified as settled juveniles. Survival declined from an initial stocking of 40,000 eggs to 181 individuals at 76 dph, corresponding to an overall survivorship of 0.45%. Previously reported survivorship among yellow tang cohorts reared to settlement at the Oceanic Institute range from 0.5–1.3% (Holt et al., 2017; Callan et al., 2018).

### DNA amplification and sequencing

DNA yield ranged from below the limit of detection (0.001 ng µL^−1^) to 280 ng µL^−1^ across 46 samples (Table S1). All samples produced PCR amplification products and subsequent 16S rRNA gene amplicon sequence data (Table S1). Following quality control, 62,681 ± 25,962 reads were recovered per sample and retained for downstream analyses (Table S1). Across the 46 samples, ASVs affiliated with the domain Bacteria dominated the dataset (1,495 bacterial *vs.* 16 archaeal ASVs), with 246 genera assigned to 23 phyla.

### Temporal trends in the development of the yellow tang microbiome

**Figure 3:**
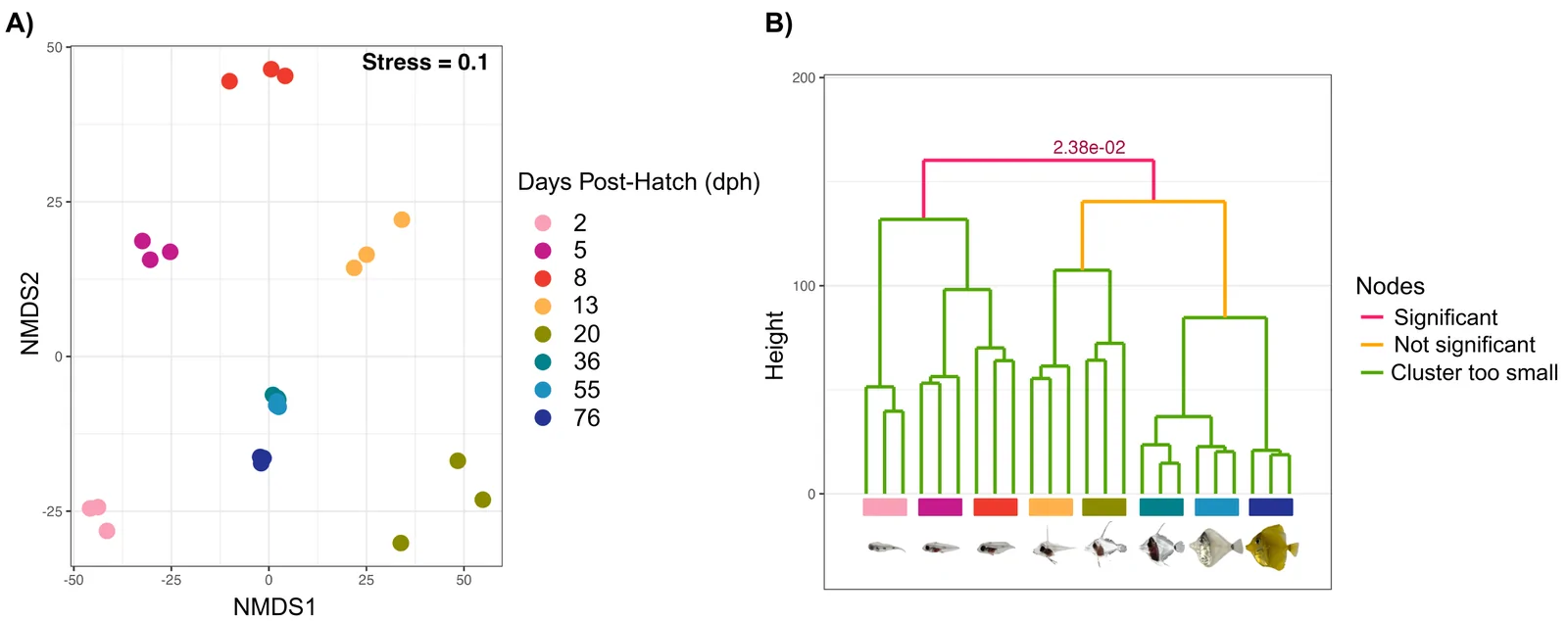
Ordination and hierarchical clustering of larval yellow tang samples from 2–76 days post-hatch (dph). (A) Non-metric multidimensional scaling (NMDS) ordination generated using Aitchison’s distances among larval microbiomes. Each point represents a sample, with color indicating timepoint sampled from 2 to 76 dph. (B) Dendrogram of larval microbiomes from hierarchical clustering with Aitchison’s distances. Pink branches indicate a significant grouping (*p* < 0.05), yellow branches non-significant grouping (>0.05), and green branches were not tested for significance due to an insufficient number of samples. Color bars and corresponding larval image at the base of the dendrogram signify the sampling timepoint of each node (*n* = 3 per timepoint).

Non-metric multidimensional scaling (NMDS) ordination showed tighter clustering among replicate larval microbiomes within the same developmental stage than among samples from different stages (Fig. 3A). A notable exception was the overlap between microbiomes from 36 and 55 dph (Fig. 3A). A multivariate analysis performed with timepoint and sample type as factors found that timepoint was significant (*p* = 0.001) and explained 46.3% of overall variability. Hierarchical clustering was then used to statistically compare individual timepoints. This analysis revealed two distinct phases in the larval period, early (2–8 dph) and late (13–76 dph), which differed significantly based on microbial composition (*p* = 0.038) (Fig. 3B).

**Figure 4:**
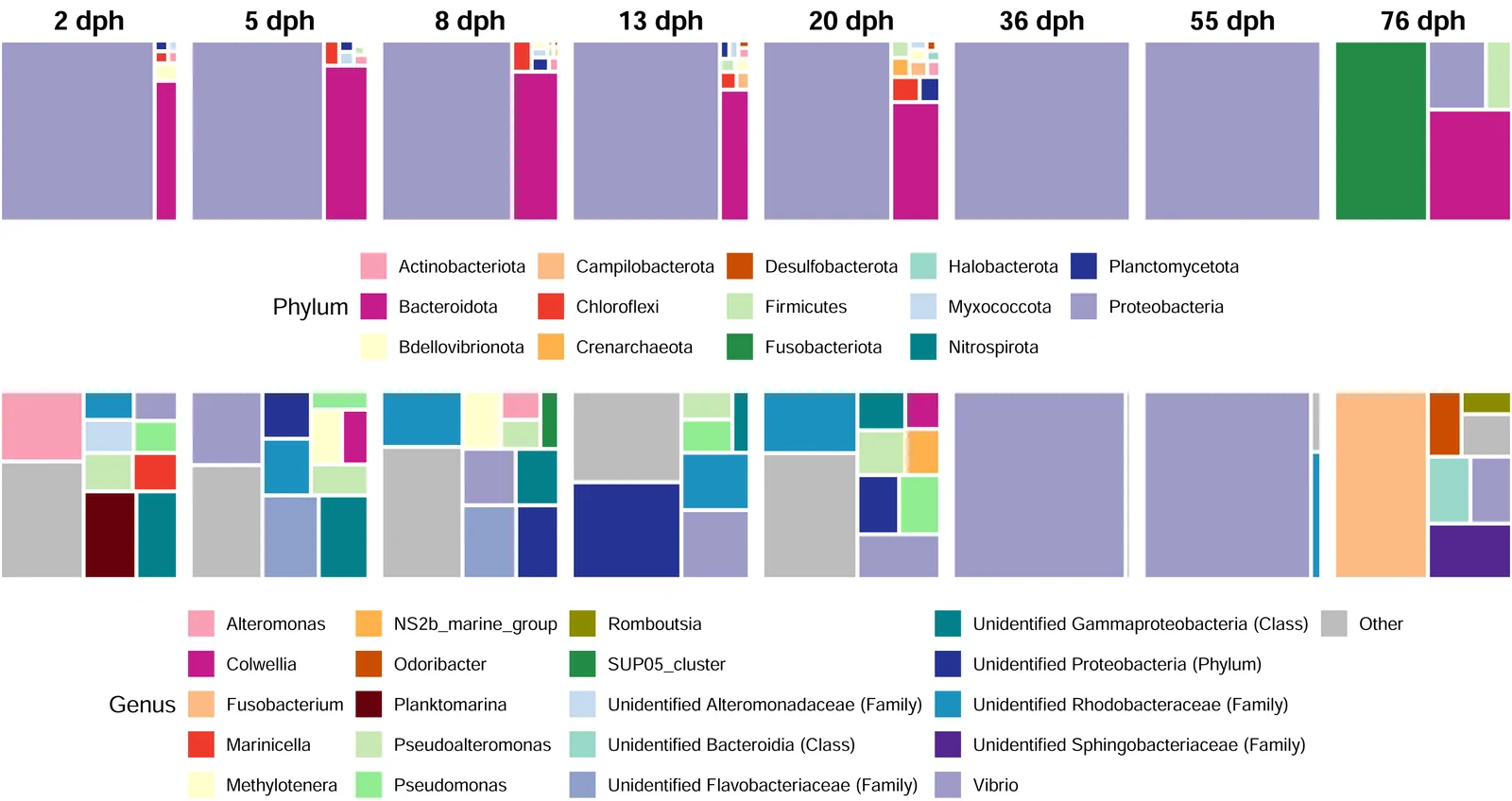
Temporal dynamics of yellow tang larval gut microbiome 2–76 days post-hatch (dph). Treemaps show relative abundance at (A) phylum and (B) genus taxonomic levels. Each square represents one of eight timepoints sampled across 2 to 76 days post-hatch larvae. The size of each tile is proportional to the mean relative abundance of each taxon across replicates (*n* = 3) at each developmental stage. For panel B, genera > 3% of the relative abundance plotted (genera < 3% grouped into “other” category). If genus was not able to be resolved, the next most specific taxonomic classification provided.

Changes in the larvae-associated microbiota from 2 to 76 dph were further examined at the phylum (Fig. 4A) and genus (Fig. 4B) levels. The phylum Proteobacteria was ubiquitous across larval samples and was the most abundant phylum at 7 of 8 timepoints, comprising 12.4–99.8% of the microbial community. In contrast, several taxa exhibited stage-specific distributions. Fusobacteriota was below detection limits from 2 to 55 dph but accounted for 52.7% of the relative abundance at 76 dph. Similarly, Firmicutes comprised <1% of the relative abundance between 2 and 55 dph but increased to 5.6% at 76 dph. At the genus level, early larval stages (2–20 dph) were characterized by a high proportion (24.5–35.8%) of low abundance ASVs grouped into the “other” category. The gammaproteobacterial genus *Vibrio* was present at all developmental stages, with relative abundances ranging from 3.9 (2 dph) to 97.2% (36 dph).

**Figure 5:**
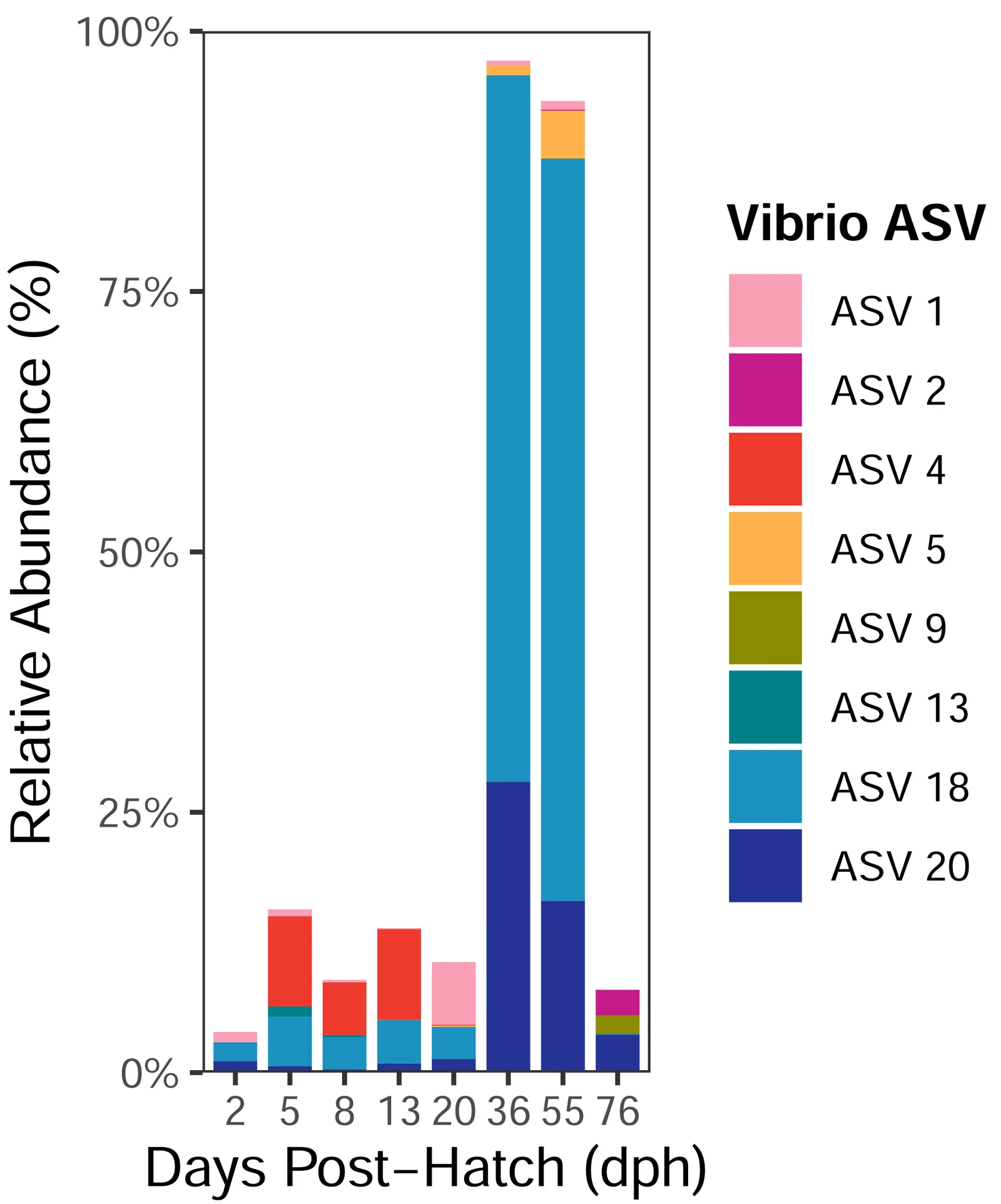
Relative abundance of *Vibrio* amplicon sequence variants (ASV) across larval development. Colors represent *Vibrio* ASVs comprising >1.5% of the mean relative abundance for any timepoint (8 ASVs total). Bars show mean *Vibrio* relative abundance across larval replicates (*n* = 3) at each sampling timepoint.

Given the presence of *Vibrio* at all timepoints we further examined its diversity and abundance. Across the dataset, 21 *Vibrio* ASVs were identified, of which eight exceeded 1.5% relative abundance at one or more of the eight sampling events. In larval samples, both the composition and relative abundance of these ASVs shifted over development from 2 to 76 dph (Fig. 5). During the early larval stages (2–20 dph), *Vibrio* ASVs 1, 4, 13, 18, and 20 were consistently present at relatively lower abundances (3.9–13.8%). At 36 and 55 dph, *Vibrio* relative abundance increased sharply (to 97.2% and 94.2%, respectively), driven primarily by ASVs 18 and 20, which correlated with the addition of live *Artemia* to their diet. By the final timepoint (76 dph), *Vibrio* abundance had returned to relative abundance levels comparable to early development (8.3%) and included the appearance of two previously undetected ASVs, 2 and 9.

A phylogenetic analysis of the eight most abundant *Vibrio* ASVs was conducted to clarify evolutionary relationships within the group (Fig. S1). The most abundant *Vibrio* ASV identified in this study (ASV 18) showed 100% V4 sequence similarity to a clade containing several described *Vibrio* species including *V. vulnificus*, *V. campbellii*, *V. coralliilyticus, V. neptunius*, *V. ostreae,* and *V. intestinalis* (Fig. S1). Similarly, ASV 20 shared an exact sequence with multiple pathogenic *Vibrio* species within the Harveyi clade (Lin et al., 2010; Goudenège et al., 2014) including *V. alginolyticus* and *V. harveyi* (Fig. S1).

Finally, we investigated the core microbiome across all larval stages during the study period. The core microbiota is commonly defined as taxa that are present in most individuals or samples across populations (Sharon et al., 2022). Here, core ASVs were defined as those occurring in at least 75% of larval samples across all eight sampling timepoints, with a minimum detection threshold of 0.05%. Under these criteria, only seven ASVs were classified as core, including three from the gammaproteobacterial genus *Vibrio* (ASVs 1, 18, and 20), two from the alphaproteobacterial family Rhodobacteraceae, one from family Ardenticatenaceae, and one from genus *Pseudoalteromonas*.

### Evaluation of sample type dissimilarity

Sample type (larvae, food stock, tank particulate matter, and rearing water) accounted for a significant but more modest proportion of the overall microbial community variation than timepoint (*R*^2^ = 0.096, *p* = 0.001), indicating that microbial communities were more compositionally similar within sample types than between them. Pairwise comparisons assessing sample type dissimilarity revealed that larval microbiomes differed significantly from rearing water (*p* = 0.04) and food stock (*p* = 0.04), but were not significantly different from tank particulate matter microbiomes (Table 1). Ordination and clustering analyses echoed these findings, showing a closer grouping of larval replicates with tank particulate matter samples throughout the larval period (Fig. S2). Examination of *Vibrio* ASVs in food, water, and particulate samples revealed that the genus was consistently higher in relative abundance in feed-associated samples (tank particulate matter, 26.7% ± 21.1%; food stock, 52.1% ± 39.9%) compared to rearing water (9.7% ± 12.2%) (Fig. S3). In food-derived sequences, ASVs 1, 4, 18, and 20 were present consistently in the early developmental periods (2–20 dph). At 36 and 55 dph, analogous to larval-derived sequences, a sharp increase in *Vibrio* relative abundance was observed in both food sample types, primarily driven by ASVs 18 and 20. In food stock only, ASV 20 remained elevated through 76 dph.

**Table 1:** Summary of pairwise comparisons. Pairwise PERMANOVA comparisons of microbial community composition among sample types. *P*-values adjusted for multiple comparisons using false discovery rate (FDR) correction. Significant *P*-values (*P* < 0.05) are denoted in bold.

Variable	R^2^	*P*-value^*^
Larvae *vs.* Water	0.06	**0.042**
Larvae *vs.* Tank Particulate Matter	0.034	0.424
Larvae *vs.* Food Stock	0.063	**0.045**
Water *vs.* Tank Particulate Matter	0.064	0.502
Water *vs.* Food Stock	0.138	**0.042**
Tank Particulate Matter *vs.* Food Stock	0.124	**0.045**

**Notes.**

* *P*-values were calculated from a distribution of 999 permutations post Bonferroni correction.

## Discussion

Culturing high-quality juveniles remains a critical bottleneck in marine ornamental aquaculture, particularly for pelagic-spawning species such as yellow tang (Moorhead & Zeng, 2010; Tlusty, 2002; Wabnitz et al., 2003). Increasing evidence indicates that unfavorable host-microbe interactions are a major driver of mass mortality in captive-reared organisms, particularly during the larval stage (Villamil et al., 2003; Ransangan & Manin, 2010; Vadstein et al., 2013; Yang et al., 2022). Intensive aquaculture systems, which typically sustain higher organic loads and stocking densities than natural environments, are prone to episodic outbreaks of opportunistic pathogens (Sanches-Fernandes, Sá-Correia & Costa, 2022). Moreover, because newly hatched larvae lack an established gut microbiota and possess immature immune defenses, they are particularly susceptible to pathogen infection (Auclert, Chhanda & Derome, 2024). Peak mortality is frequently concurrent with the first feeding and gut maturation, yet the role of the larval microbiome in survivorship during this transition and the duration of the larval period remains poorly understood. Improved understanding of gut microbiota assembly and early establishment of a beneficial gut microbiota may therefore be critical for advances in larval survival and development (Auclert, Chhanda & Derome, 2024).

Model organisms, such as zebrafish (*Danio rerio*), and farm-raised fish have provided foundational insights into early-life microbiota assembly (Luan et al., 2023); however, these findings may not translate to marine ornamental fish species which have unique physiological and life history traits that make captive-rearing challenging (Holt et al., 2017; Olivotto et al., 2005). Consequently, the evolution of the larval microbiome in an aquaculture setting is largely unexplored for these species. In collaboration with the Oceanic Institute, we leveraged access to the only commercial-scale production of yellow tang - one of the most highly demanded marine ornamental species in the aquarium trade (Williams et al., 2009; DLNR, 2015). The findings here provide an opportunity to examine microbiome dynamics in a pelagic-spawning marine ornamental species under commercial aquaculture conditions over the course of larval development.

While marine ornamental species present unique rearing challenges, the larval yellow tang exhibited similar trends in microbiota assembly and composition compared with other farm-raised fish species, suggesting conserved patterns in early-life microbiota assembly. The most abundant phyla identified in our study across time points were Proteobacteria, Bacteroidota, Fusobacteriota, and Firmicutes. A recent review of 24 farm-raised fish microbiomes similarly reported these phyla as the most dominant across diverse fish species (Kanika et al., 2025). Of these phyla, Proteobacteria was the most dominant identified in our larval samples. Although frequently detected in fish gut microbiomes (Huang et al., 2020), elevated abundances may indicate dysbiosis, as this group includes many opportunistic pathogens (Xavier, Severino & Silva, 2023; Mekasha & Linke, 2021). For example, in Yunlong grouper (*Epinephelus fuscoguttatus*), Proteobacteria was significantly elevated within the gut microbiome of diseased individuals *versus* their healthy counterparts (Ma et al., 2019). Moreover, in grass carp (*Ctenopharyngodon idellus*), Proteobacteria became the dominant phylum under starvation conditions (Tran et al., 2018). The dominance of Proteobacteria at seven of the eight sampling timepoints in our study may suggest a potential state of dysbiosis in the larval microbiome prior to settlement.

We observed a distinct shift in the microbiome during the final timepoint (76 dph) from a Proteobacteria-dominated microbiome to domination by Fusobacteriota and Bacteroidota, along with a marked increase in Firmicutes, which was not concurrent with any dietary changes. Firmicutes and Bacteroidota are frequently described as abundant and beneficial members of marine fish gut microbiomes (Huang et al., 2020; Talwar et al., 2018). Fusobacteriota has previously been reported to be acquired late in the larval cycle of the zebrafish microbiome (Stephens et al., 2016) which may reflect a functional role for this symbiont that becomes important after the larval phase. In grass carp, Li et al. (2024) reported inverse co-occurrence patterns between Proteobacteria-associated genera and members of Fusobacteriota, Firmicutes, and Bacteroidota, supporting the interpretation that these taxa form two distinct ecological groups within the microbiome. Furthermore, metagenomic analysis of functional genes revealed that Proteobacteria (Group 1) encoded fewer genes associated with carbohydrate degradation, but a greater abundance of virulence factors and antibiotic resistance genes compared to Fusobacteria/Firmicutes/Bacteroidetes (Group 2) (Li et al., 2024). This analysis defined two functional groups, and the ratio of Group 2: Group 1 was proposed as a potential biomarker, with higher ratios indicative of improved fish health. This ratio increased in yellow tang upon settlement, which is a period associated with lower mortality and increased population stability in an aquaculture setting (Holt et al., 2017).

At the community level, we observed the larval yellow tang microbiome to be highly dynamic, with substantial restructuring occurring over timescales of days to weeks. Ordination and multivariate analyses consistently indicated that microbiomes associated with the same developmental stage were more similar to one another than to those from other stages, while hierarchical clustering divided the larval stage into two distinct periods (2–8 dph and 13–76 dph). Together, these findings support ontogeny as a primary driver of microbiome composition. We note, however, that microbiomes from the earliest developmental stages (2–20 dph) were derived from whole-larvae homogenates due to the inability to isolate gut tissue at these stages as previously described (Wilkes Walburn et al., 2018). Therefore, these samples may include microbial contributions from non-gut tissues and influence the temporal patterns observed. Hierarchical clustering did not show grouping associated with the transition from whole-larvae to dissected-gut samples, and homogenate microbiomes were distributed across the most pronounced clusters.

Rapid restructuring of the gut microbiome during early life, along with unique microbiota signatures associated with different developmental stages, has been echoed in other marine teleosts (Stephens et al., 2016) and in mammals (Stewart et al., 2018). Our results indicate that larval yellow tang exhibit a similar pattern of early-life microbiome instability consistent with an underdeveloped innate immune system and limited ability of the host to modulate emerging symbiotic associations. In contrast, adult vertebrates typically harbor more stable gut microbiomes, although other factors such as diet and disease remain influential (Sieler Jr et al., 2023). Extending microbiome characterization through the juvenile and adult stages will be important for determining whether stabilization occurs in yellow tang post-settlement.

We also considered the effects of the microbiome of food particles, the tank water and tank particulate matter on the yellow tang microbiome. Although timepoint was the strongest driver of community structure, pairwise comparisons among sample types showed that larval microbiomes differed significantly from rearing water and food stock communities, but not from tank particulate matter. This suggests that the larval microbiome undergoes structured developmental succession while remaining closely associated with the particle-associated microbial pool in the rearing environment. Our NMDS ordination indicated structured shifts in bacterial community composition across yellow tang larval development, with samples clustering more strongly by days post-hatch than by sample type. Although some overlap was observed among larvae, rearing water, food stock, and tank particulate matter, early timepoints occupied distinct regions of the ordination relative to later stages, suggesting temporal succession in the larval rearing microbiome. This pattern suggests that the larval microbiome is not simply a passive reflection of the surrounding water or food source, but may be more closely linked to the particle-associated microbial pool. Tank particulates likely represent a mixed compartment that may include uneaten feed, microbial aggregates, biofilm fragments, larval-associated detritus, and fecal material. Therefore, the similarity between larval and particulate microbiomes could reflect bidirectional microbial exchange, with larvae both acquiring bacteria from particle-associated communities and contributing gut-associated bacteria to the particulate pool through fecal shedding or sloughed material. Together, these results suggest that yellow tang larval microbiome development is shaped primarily by ontogenetic stage, while tank particulate matter may serve as an important reservoir or integrative signal of larval-associated microbial communities. Rapid restructuring of the gut microbiome post-feeding has been found in other marine ornamental species like the clownfish (*Amphiprion ocellaris*) (Parris, Morgan & Stewart, 2019), and diet-driven modulation of the gut microbiota has been documented across a broad range of fish including tilapia (Wang et al., 2022), zebrafish (*Danio rerio*) (Stephens et al., 2016; Sieler Jr et al., 2023), yellowtail kingfish (*Seriola lalandi*) (Wilkes Walburn et al., 2018), European sea bass (*Dicentrarchus labrax*), and gilthead seabream (*Sparus aurata*) (Najafpour et al., 2023).

We next assessed the relationship of feed and temporal patterns in the relative abundance of *Vibrio*. *Vibrio* species are common pathogens in aquaculture that cause vibriosis across a wide range of cultured fish, often leading to severe hemorrhagic septicemia, skin ulcers, and high mortalities, posing a significant threat to fish health and production sustainability (Vandeputte et al., 2024). Here, *Vibrio* was ubiquitous across all developmental stages but varied dramatically in relative abundance (3.9–97.2%). The increase in *Vibrio* relative abundance observed at 36 and 55 dph correlating with the addition of enriched *Artemia* to the larval diet suggests this food source may be a vector driving *Vibrio* enrichment in the gut. This is consistent with cultures of live feed like *Artemia* being well-established reservoirs for diverse marine bacteria, including multiple pathogenic *Vibrio* species (Dhont et al., 2013; Gómez-León et al., 2005; Sanches-Fernandes, Sá-Correia & Costa, 2022). Moreover, elevated *Vibrio* abundance within rotifer and *Artemia* cultures have been previously reported at the Oceanic Institute (Forbes, 2016), and live prey has similarly been identified as the predominant source of *Vibrio* and other potential pathogens in European sea bass and gilthead sea bream hatcheries (Najafpour et al., 2023). While other factors like temperature fluctuations and high larval density also impact *Vibrio* abundance in an aquaculture setting, we conclude these are less likely drivers of the *Vibrio* variability observed in this study. With continuous water exchange and daily monitoring, temperature and other water quality parameters did not vary significantly with larval age, and incidental mortality resulted in a decreasing larval density across the study period. However, increasing feed intensity of dry feed and *Artemia* with age warrants further investigation to determine whether elevated *Vibrio* abundance is driven by the introduction of enriched *Artemia* or by feed density thresholds.

Phylogenetic analysis of the eight most abundant *Vibrio* ASVs identified in this study found that ASV 20 shared an identical V4 region sequence with several pathogenic *Vibrio* species, including *V. alginolyticus* and *V. harveyi* (Gómez-León et al., 2005; Zhang, He & Austin, 2020), that are known to be associated with *Brachionus* and *Artemia* cultures (Sanches-Fernandes, Sá-Correia & Costa, 2022). While 16S rRNA gene sequence data cannot definitively resolve species identity, this relationship highlights the possibility that *Vibrio* may contribute to larval yellow tang mortality. These findings collectively support that feed shapes microbiome composition and that live prey, in particular, may serve as a vector of exposure to *Vibrio*-associated microbial communities during early development.

Consistent with a dynamically structured microbiome, only a small number of core taxa were detected across larval samples. Of the seven core ASVs detected, three belonged to the genus *Vibrio*. Previous studies in mice (Wang et al., 2019) and zebrafish (Roeselers et al., 2011) have suggested that consistently present taxa may reflect important host-microbe symbioses. In zebrafish, for example, Roeselers et al. (2011) identified 21 shared operational taxonomic units (OTUs) across individuals raised in different facilities and captured from the wild. In contrast, the paucity of persistent taxa observed here once again suggests that host-mediated selection and maintenance of gut microbiota may be limited during the early larval stage of yellow tang in an aquaculture setting. Moreover, based on our phylogenetic analysis of the *Vibrio* ASVs, including the three found to be core ASVs, we hypothesize that the persistence of these ASVs is more likely to be the result of repeat exposure to live feed as opposed to a host-mediated symbiosis.

As interest grows in microbiome-based approaches to improve aquaculture production, we consider whether probiotics may support larval survival and health in yellow tang, including the probiotic feed additive Bactocell employed in this study. Application of probiotics, primarily through water or feed supplementation, has been shown to enhance fish survival and growth (Llewellyn et al., 2014) and act as a mechanism of disease control in an aquaculture setting (Hoseinifar et al., 2018), with *Lactobacillus*, *Lactococcus, Bacillus*, and *Pediococcus* among the most commonly utilized genera within finfish aquaculture (Rwezawula et al., 2025). However, successful colonization of introduced probiotic strains remains variable, as these microorganisms must compete with established microbial communities and be delivered at sufficient densities to exert meaningful effects (Giatsis et al., 2016; Wuertz, Schroeder & Wanka, 2021). Consistent with these challenges, we did not detect the bacterium *P. acidilactici,* the primary strain within the feed additive Bactocell, in larval, food, or water samples at any time point indicating this bacterium was below our detection threshold or colonization was unsuccessful. This finding underscores a growing recognition that exogenous probiotic strains may face inherent limitations in establishing within host microbiomes and their environment (Wuertz, Schroeder & Wanka, 2021; Rwezawula et al., 2025). Moreover, identification of probiotic candidates isolated directly from the host digestive system, rearing water, or rearing tank itself, have been recently gaining attention as a promising strategy as these strains are more likely to persist at biologically relevant abundances (Ringø et al., 2014; Rwezawula et al., 2025). A compelling selection criterion for such candidates is the capacity to disrupt quorum sensing in pathogenic bacteria, which is a primary mechanism by which pathogens regulate virulence gene expression and antimicrobial resistance (Rwezawula et al., 2025). Given the persistent challenge of high larval mortality in yellow tang and the elevated *Vibrio* abundances identified in this study, this strategy among others are worthy areas of future study to pursue for yellow tang and other pelagic spawning ornamental fish aquaculture.

## Conclusions

Understanding and managing host-microbiota interactions is essential for improving commercial production of high-demand ornamental species (Perry et al., 2020; Auclert, Chhanda & Derome, 2024). Here, we characterized the microbiome assembly in larval yellow tang during a commercial production run with survival rates representative of current industry standards. Our results demonstrate that the larval microbiome is consistently dominated by Proteobacteria, but also exhibits high temporal variability with some taxa characteristic of particular developmental stages. The increased relative abundance of *Vibrio* coincident with the introduction of enriched *Artemia* suggests that live feed cultures may be a key vector of this genus. Though live feeds are a critical component to marine teleost husbandry due to their size ranges and high nutritional value (Sanches-Fernandes, Sá-Correia & Costa, 2022), their role in the introduction of potential pathogens should be further investigated.

We propose that the prevalence of potentially pathogenic *Vibrio* species, combined with an immature and unstable host microbiome, contributes to the high mortality rates characteristic of the larval stage. Earlier establishment of a beneficial host microbiota may therefore enhance larval resistance to episodic pathogen exposure. Improving our understanding of microbiome dynamics in captive-reared yellow tang is a critical step toward increasing juvenile production, supporting sustainable aquarium trade practices, and reducing pressure on wild populations of this ecologically and economically important species.

## Supplemental Information

10.7717/peerj.21690/supp-1 Supplemental Information 1 Phylogenetic analysis illustrating relationships among the hypervariable region V4 of the 16S rRNA gene from taxonomically described *Vibrio* and ASVs recovered in this study The scale bar corresponds to 0.05 substitutions per nucleotide position. ASVs > 1.5% of the total relative abundance color coded.

10.7717/peerj.21690/supp-2 Supplemental Information 2 Ordination of larval, food stock, tank particulate matter, and water from 2–76 days post-hatch (dph) Non-metric multidimensional scaling (NMDS) ordination generated using Aitchison’s distances among all samples. Each point represents a sample, with color indicating timepoint sampled from 2 to 76 dph and shape indicating sample type. Ellipses group all samples from the same timepoint.

10.7717/peerj.21690/supp-3 Supplemental Information 3 Relative abundance of *Vibrio* amplicon sequence variants (ASV) across larval, food stock, tank particulate matter, and water samples Colors represent *Vibrio* ASVs comprising >1.5% of the mean relative abundance for any timepoint (7 ASVs total). Bars show mean *Vibrio* relative abundance across replicates (*n* = 3) for each sample type at each sampling timepoint.

10.7717/peerj.21690/supp-4 Supplemental Information 4 R code R script imports the QIIME2-exported ASV table, taxonomy, phylogenetic tree, and sample metadata into a phyloseq object, quality-filters the data, and transforms counts to relative or CLR abundances. It then generates the figures produced from our analyses (treemaps, NMDS ordinations, a clustering dendrogram, and Vibrio abundance plots) and runs the associated statistical tests, including PERMANOVA.

10.7717/peerj.21690/supp-5 Supplemental Information 5 QIIME2 code QIIME2 pipeline processes raw paired-end 16S rRNA sequences (EMP V4 primers) to generate amplicon sequence variants (ASVs), which are then taxonomically classified using the SILVA database and quality-filtered. The final filtered ASV table, taxonomy assignments, representative sequences, and rooted phylogenetic tree were then exported for downstream analysis in RStudio.

10.7717/peerj.21690/supp-6 Supplemental Information 6 Sample metadata and sequencing summary

## Additional Information and Declarations

### DNA Deposition

The following information was supplied regarding the deposition of DNA sequences: The raw sequence data are available at NCBI BioProject: PRJNA1184414.

### Data Availability

The following information was supplied regarding data availability: The code and data are available in the Supplemental Files. The code is available at GitHub and Zenodo: - https://github.com/AnnieDeck/Yellow-Tang. - Deck, A. (2026). YT_PeerJ_Data_2026. In PeerJ. Zenodo. https://doi.org/10.5281/zenodo.21694648. The raw sequence data are available at NCBI BioProject: PRJNA1184414.
