# Supplementary material for: Succession of the yellow tang (Zebrasoma flavescens) microbiome from hatch to settlement in a recirculating aquaculture system
Source: PeerJ. 2026 Sep 21;14:e21690. doi: 10.7717/peerj.21690 (PMC13603922; doi:10.7717/peerj.21690)
Supplement: Supplementary file 1 [file peerj-14-21690-s001.pdf]

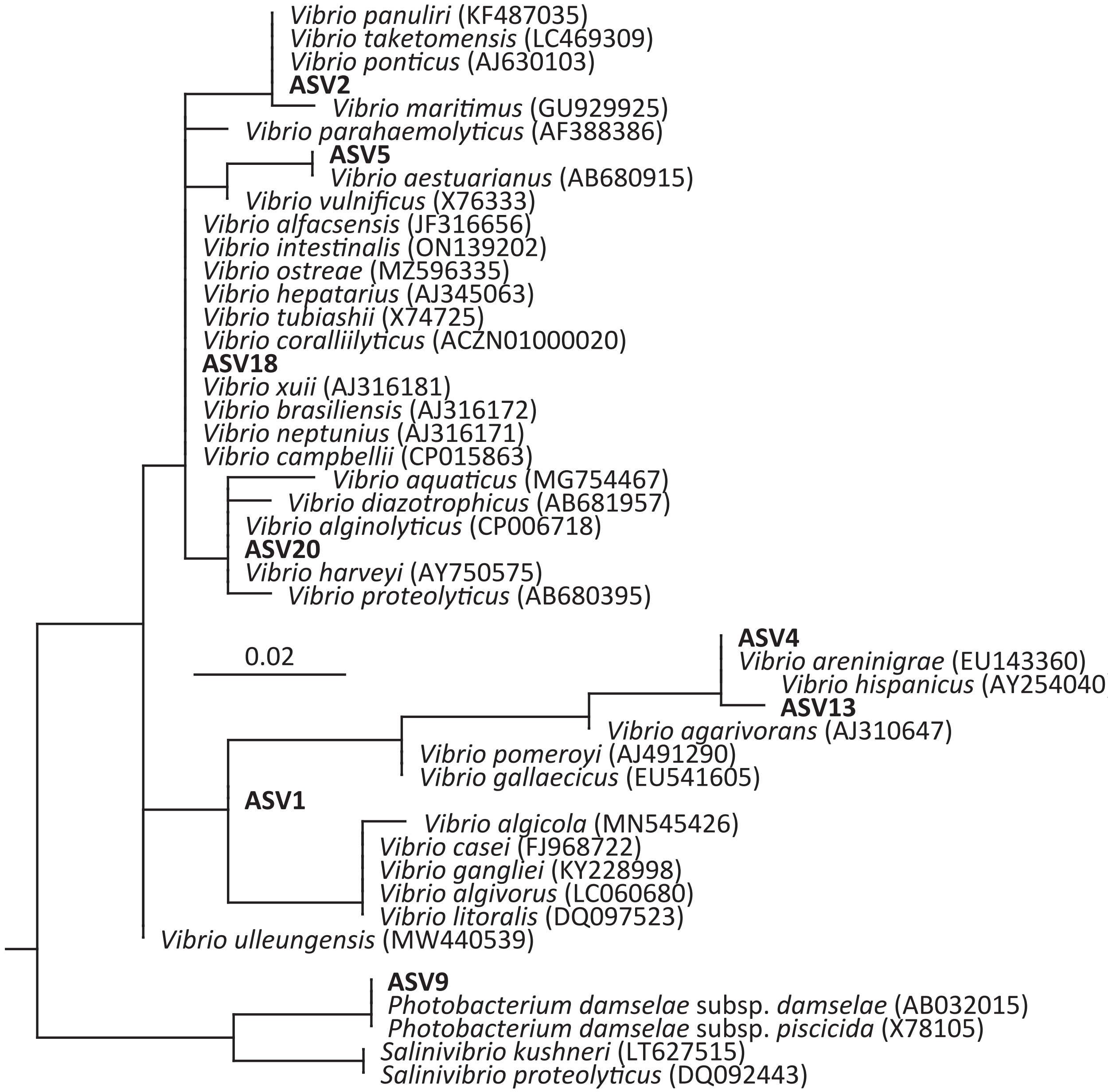

Vibrio panuliri (KF487035)
Vibrio taketomensis (LC469309)
Vibrio ponticus (AJ630103)
ASV2
Vibrio maritimus (GU929925)
Vibrio parahaemolyticus (AF388386)
ASV5
Vibrio aestuarianus (AB680915)
Vibrio vulnificus (X76333)
Vibrio alfacsensis (JF316656)
Vibrio intestinalis (ON139202)
Vibrio ostreae (MZ596335)
Vibrio hepatarius (AJ345063)
Vibrio tubiashii (X74725)
Vibrio coralliilyticus (ACZN01000020)
ASV18
Vibrio xuii (AJ316181)
Vibrio brasiliensis (AJ316172)
Vibrio neptunius (AJ316171)
Vibrio campbellii (CP015863)
Vibrio aquaticus (MG754467)
Vibrio diazotrophicus (AB681957)
Vibrio alginolyticus (CP006718)
ASV20
Vibrio harveyi (AY750575)
Vibrio proteolyticus (AB680395)
0.02
ASV4
Vibrio areninigrae (EU143360)
Vibrio hispanicus (AY254040)
ASV13
Vibrio agarivorans (AJ310647)
Vibrio pomeroyi (AJ491290)
Vibrio gallaecicus (EU541605)
ASV1
Vibrio algicola (MN545426)
Vibrio casei (FJ968722)
Vibrio gangliei (KY228998)
Vibrio algivorus (LC060680)
Vibrio litoralis (DQ097523)
Vibrio ulleungensis (MW440539)
ASV9
Photobacterium damselae subsp. damselae (AB032015)
Photobacterium damselae subsp. piscicida (X78105)
Salinivibrio kushneri (LT627515)
Salinivibrio proteolyticus (DQ092443)
