# Supplementary material for: Succession of the yellow tang (Zebrasoma flavescens) microbiome from hatch to settlement in a recirculating aquaculture system
Source: PeerJ. 2026 Sep 21;14:e21690. doi: 10.7717/peerj.21690 (PMC13603922; doi:10.7717/peerj.21690)
Supplement: Supplementary file 2 [file peerj-14-21690-s002.pdf]

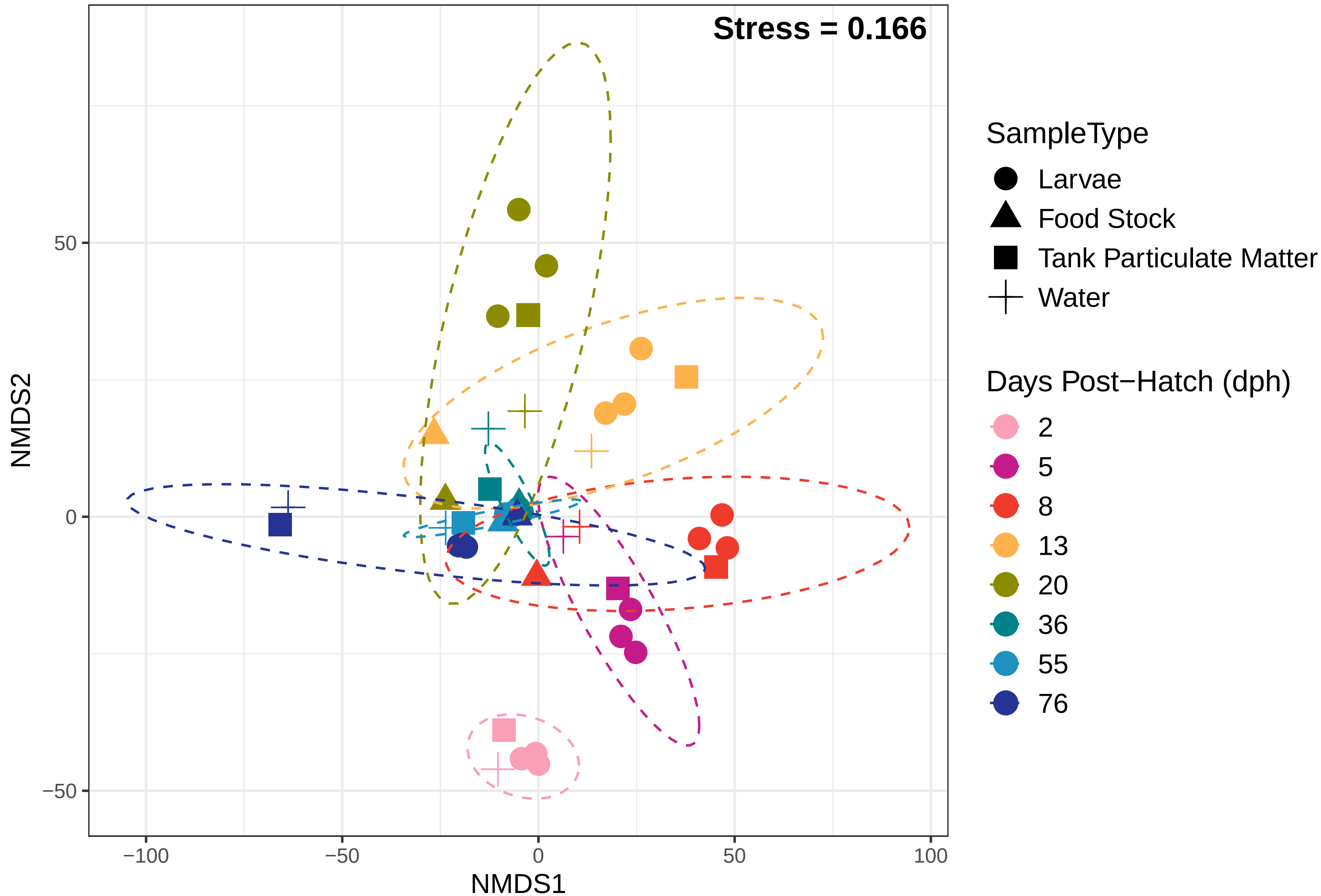

Stress = 0.166
NMDS2
NMDS1
50
0
−50
−100
−50
0
50
100
SampleType
Larvae
Food Stock
Tank Particulate Matter
Water
Days Post−Hatch (dph)
2
5
8
13
20
36
55
76
