# Supplementary material for: Succession of the yellow tang (Zebrasoma flavescens) microbiome from hatch to settlement in a recirculating aquaculture system
Source: PeerJ. 2026 Sep 21;14:e21690. doi: 10.7717/peerj.21690 (PMC13603922; doi:10.7717/peerj.21690)
Supplement: Supplementary file 3 [file peerj-14-21690-s003.pdf]

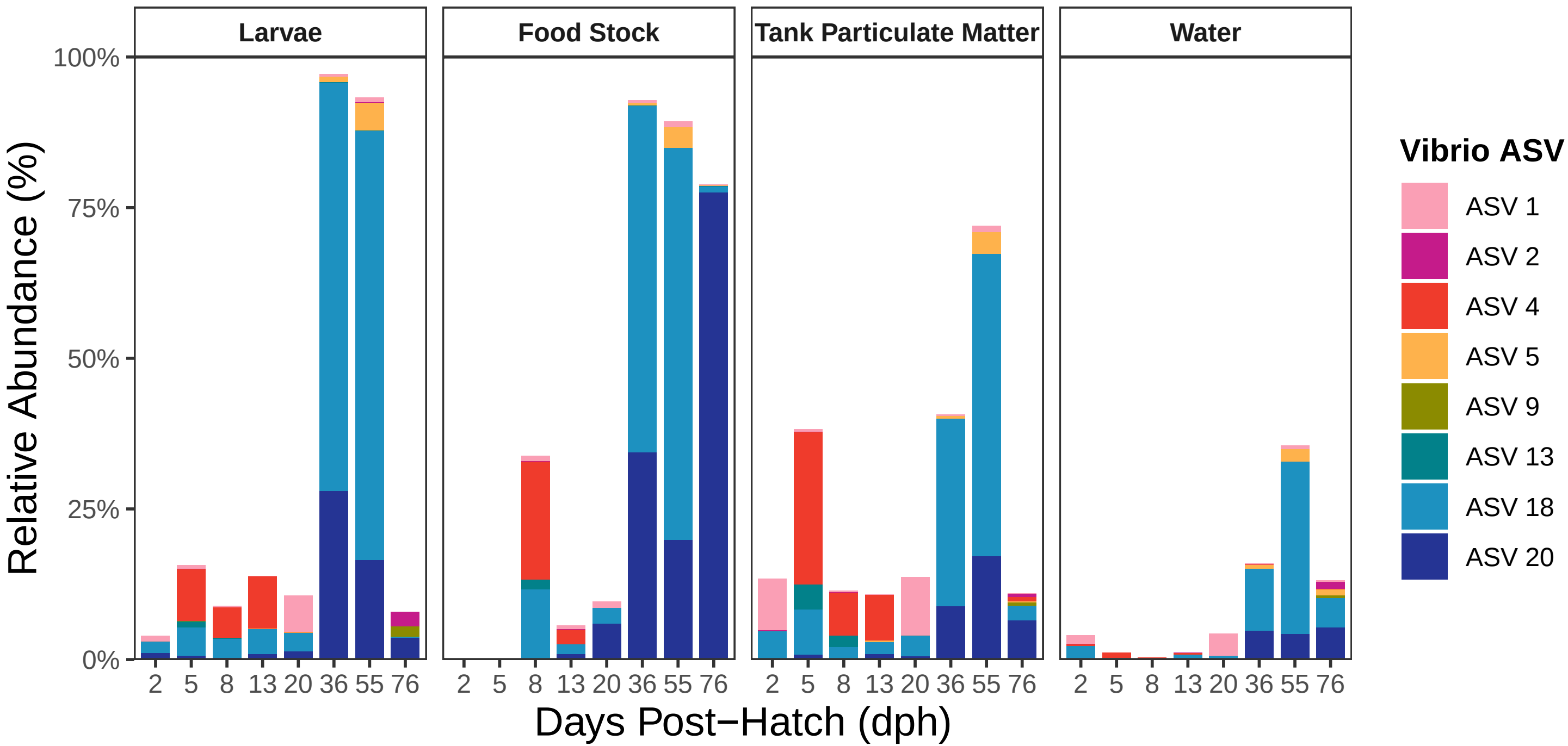
Larvae
Food Stock
Tank Particulate Matter
Water
Relative Abundance (%)
100%
75%
50%
25%
0%
2
5
8
13
20
36
55
76
Days Post−Hatch (dph)
Vibrio ASV
ASV 1
ASV 2
ASV 4
ASV 5
ASV 9
ASV 13
ASV 18
ASV 20
